# Sir Francis Bacon’s Cipher Story

**Published:** 1895-07

**Authors:** 


### BOOK NOTICES.

Sir Francis Bacon's Cipher Story, discovered and deciphered by Orville W. Owen, M. D., Book V. Detroit: Howard Publishing Co., 1895. Paper cover. Price, 50 cents.

The readers of this journal have from time to time read notices of this astonishing work, as the various volumes have come out. For the convenience of those who may wish to refer to these various notices, it may be said that they may be found as follows: June, 1894, pages 163 and 186; September, 1894, page 290; May, 1895, page 246.

The present volume relates the history of Bacon's life at the Court of France; how he met the King of Navarre and confessed to him his heirship to the throne of England; how Navarre sought to escape from the Court of the King of France, by whom he was suspected of intention to raise a rebellion in the interest of the Huguenots, and so was kept a virtual prisoner; how he was frustrated, but succeeded later. Bacon also relates, with remarkable frankness, how he fell in love with Navarre's wife, Queen Margaret, sister of the King of France; how the Queen accepted his love, while her husband was conducting the war against the crown, which had been anticipated by the King.

The whole story is marvelous, but must be read to be appreciated. The important statement concerning this volume should here be made that it was deciphered entirely by Dr. Owen's assistants, to whom he had taught the cipher. This shows that any one can learn the cipher, and is an unanswerable proof of the truth of Dr. Owen's discovery.

